# The Potential Effects of Curcumin on Pulmonary Fibroblasts of Idiopathic Pulmonary Fibrosis (IPF)—Approaching with Next-Generation Sequencing and Bioinformatics

**DOI:** 10.3390/molecules25225458

**Published:** 2020-11-21

**Authors:** Wei-An Chang, Chia-Min Chen, Chau-Chyun Sheu, Ssu-Hui Liao, Ya-Ling Hsu, Ming-Ju Tsai, Po-Lin Kuo

**Affiliations:** 1 Graduate Institute of Clinical Medicine, College of Medicine, Kaohsiung Medical University, Kaohsiung 807, Taiwan; 960215kmuh@gmail.com (W.-A.C.); sheucc@gmail.com (C.-C.S.); s0970215575@gmail.com (S.-H.L.); kuopolin@seed.net.tw (P.-L.K.); 2 Department of Internal Medicine, School of Medicine, College of Medicine, Kaohsiung Medical University, Kaohsiung 807, Taiwan; 3 Division of Pulmonary and Critical Care Medicine, Department of Internal Medicine, Kaohsiung Medical University Hospital, Kaohsiung Medical University, Kaohsiung 807, Taiwan; kmuronald@gmail.com; 4 Graduate Institute of Medicine, College of Medicine, Kaohsiung Medical University, Kaohsiung 807, Taiwan; yainghsu@kmu.edu.tw; 5 Center for Biomarkers and Biotech Drugs, Kaohsiung Medical University, Kaohsiung 807, Taiwan; 6 Institute of Medical Science and Technology, National Sun Yat-Sen University, Kaohsiung 804, Taiwan

**Keywords:** bioinformatics, curcumin, fibroblasts, idiopathic pulmonary fibrosis, microRNA, next-generation sequencing, *KLF10*, *TIEG*

## Abstract

Idiopathic pulmonary fibrosis (IPF) is a chronic and progressive interstitial lung disease. Currently, therapeutic options are limited for this fatal disease. Curcumin, with its pleiotropic effects, has been studied for its potential therapeutic utilities in various diseases, including pulmonary fibrosis. However, the detailed mechanisms have not been studied comprehensively. We conducted a next-generation sequencing and bioinformatics study to investigate changes in the profiles of mRNA and microRNA after curcumin treatment in IPF fibroblasts. We identified 23 downregulated and 8 upregulated protein-coding genes in curcumin-treated IPF fibroblasts. Using STRING and IPA, we identified that suppression of cell cycle progression was the main cellular function associated with these differentially expressed genes. We also identified 13 downregulated and 57 upregulated microRNAs in curcumin-treated IPF fibroblasts. Further analysis identified a potential microRNA-mediated gene expression alteration in curcumin-treated IPF fibroblasts, namely, downregulated hsa-miR-6724-5p and upregulated *KLF10*. Therefore, curcumin might decrease the level of hsa-miR-6724-5p, leading to increased *KLF10* expression, resulting in cell cycle arrest in curcumin-treated IPF fibroblasts. In conclusion, our findings might support the potential role of curcumin in the treatment of IPF, but further in-depth study is warranted to confirm our findings.

## 1. Introduction

Idiopathic pulmonary fibrosis (IPF) is a chronic, progressive interstitial lung disease characterized by fibrosing interstitial pneumonitis [1,2,3,4,5,6,7,8]. The clinical symptoms are usually nonspecific, including exertional dyspnea and dry cough. Auscultation of the lungs usually reveals high-pitched, fine, Velcro-like inspiratory crackles, mainly in the bilateral basal lung fields [5]. High-resolution computed tomography is currently the key diagnostic tool, typically revealing the usual interstitial pneumonia (UIP) pattern, characterized by honeycomb cysts and reticulation predominantly involving the subpleural and basal areas, which gradually progress to the whole lungs [2,3,4]. With an estimated incidence of about 20–300 cases per 1,000,000 person-years, IPF currently affects about 3 million people worldwide [3,5,9]. Untreated IPF patients usually have poor quality-of-life related to progressively disabling dyspnea, with a median survival of around 3–5 years [3,4,10].

Although the mechanisms underlying the pathogenesis of IPF remain largely unknown, fibroblasts are recognized as key players in the pathogenesis and progression of IPF [1,6,7,11]. Increased proliferation, activation, and migration of fibroblasts, as well as their increased production of extracellular matrix components and differentiation to myofibroblasts, are all proposed as key mechanisms underlying the development and progression of IPF [3,6,7,12,13,14]. Oxidative stress and inflammation are also common contributing factors for pulmonary fibrosis [15,16,17].

The currently available treatment modalities for IPF are limited. Currently, only two medications, nintedanib and pirfenidone, are available to hamper disease progression and improve patients’ quality-of-life [1,18,19,20,21,22,23,24]. However, the treatment response to these drugs varies and currently cannot be predicted with any clinical characteristics or biomarkers. Continuous efforts are therefore made to find any potential novel therapeutic agents for IPF.

Curcumin, also known as diferuloylmethane, is the principle phenolic compound extracted from turmeric (*Curcuma longa*), a member of the ginger family (*Zingiberaceae*) [25,26]. Besides its use in food coloring and flavoring, it has been widely used in traditional Chinese and Indian medicine for centuries [25,27]. Curcumin has pleiotropic effects, including regulation of transcription factors, cytokines, adhesion molecules, and enzymes, resulting in a wide range of therapeutic properties [27]. The activities of curcumin, including antimicrobial, anti-inflammation, antioxidation, antineoplastic, antifibrotic, and immune-modulating effects, were extensively studied [25,26,27]. Due to these properties, its effects in several pulmonary diseases, including chronic obstructive pulmonary disease, asthma, lung cancer, acute respiratory distress syndrome, and pulmonary fibrosis, were previously reported [27,28,29,30,31,32,33,34].

**Figure 1:**
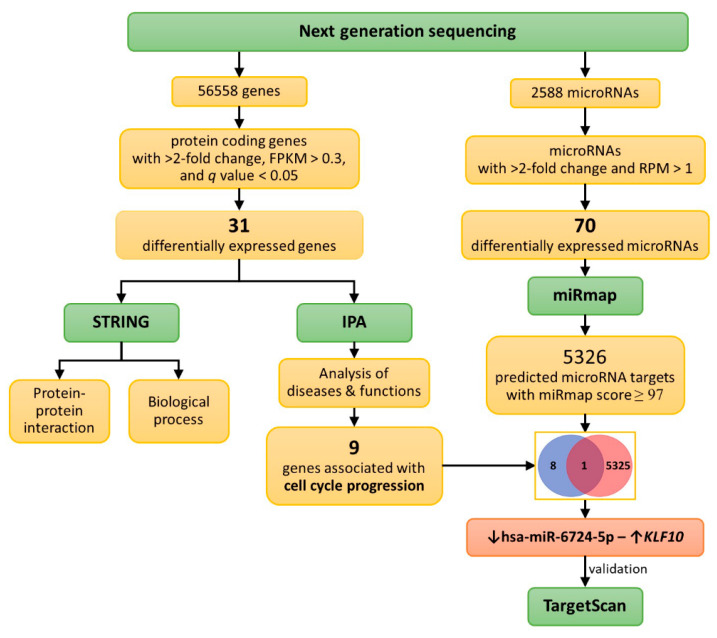
Schematic illustration of study design and analysis strategy. Idiopathic pulmonary fibrosis (IPF) fibroblasts were treated with either 10 μM curcumin or 0.1% dimethyl sulfoxide (DMSO) (control) for 48 h. Then, the harvested RNAs were sequenced using the next-generation sequencing (NGS) platform to obtain the microRNA and mRNA expression profiles. Differentially expressed protein-coding genes were selected for further bioinformatic analyses using the Search Tool for the Retrieval of Interacting Genes (STRING) database and the Ingenuity^®^ Pathway Analysis (IPA) database. The upregulated and downregulated microRNAs were analyzed with miRmap for target prediction. Potential microRNA–mRNA interactions associated with curcumin treatment were identified, and Venn diaphragm analysis was used to focus on those related to cell cycle progression. These interactions were further validated using another prediction database (TargetScan).

Some in vitro and in vivo studies showed the effects of curcumin on fibroblasts, such as promoting apoptosis, inhibiting differentiation, modulating matrix metalloproteinase production, and enhancing antioxidant defense mechanisms [25,35,36,37,38,39]. However, the effects of curcumin in modulating gene expression profiles in pulmonary fibroblasts were not systematically investigated. The current study aimed to explore the effects of curcumin on gene expression profiles, including mRNA and microRNA changes, in IPF fibroblasts using next-generation sequencing (NGS) and bioinformatic analyses (Figure 1).

## 2. Results

### 2.1. Curcumin Inhibited the Proliferation/Viability of IPF Fibroblasts

**Figure 2:**
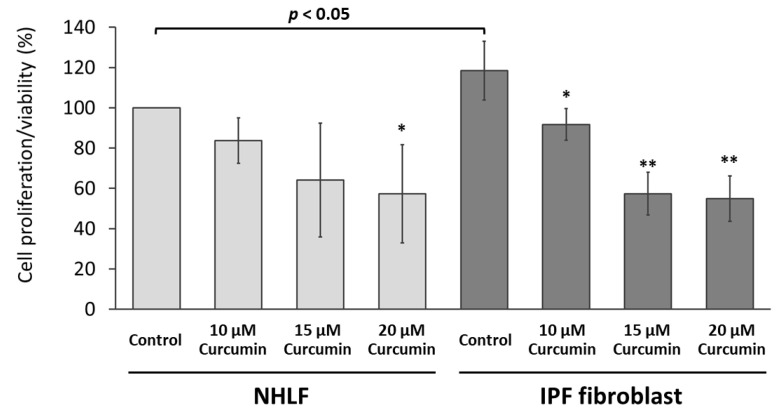
Curcumin inhibited the proliferation/viability of pulmonary fibroblasts. Primary human lung fibroblasts from a normal healthy subject (NHLF) and an idiopathic pulmonary fibrosis (IPF) patient (IPF fibroblast) were treated with either curcumin (10, 15, or 20 μM) or the carrier solvent (0.1% DMSO, the control) for 48 h. The cell proliferation/viability was assessed using the WST-1 Cell Proliferation Assay. The results of four independent experiments are presented in means ± standard deviations. The results of cells treated with the carrier solvent (control groups) were compared using the *t*-test. The results of the same cell were compared between groups using analysis of variance, followed by Dunnett’s test (* *p* < 0.05; ** *p* < 0.0001, as compared to the corresponding control group).

The IPF fibroblasts showed significantly increased cell proliferation/viability compared with normal human lung fibroblasts (Figure 2). Curcumin inhibited the proliferation/viability of both normal human lung fibroblasts and IPF fibroblasts in a dose-dependent manner, while the effect appeared more prominent in IPF fibroblasts (Figure 2). The inhibiting effect of 10 μM of curcumin was significant in IPF fibroblasts but nonsignificant in normal human lung fibroblasts. The inhibiting effect of 15 or 20 μM of curcumin on cell proliferation/viability was so great that an insufficient number of viable cells was available to send for NGS. Based on this testing result and previous studies evaluating the effects of curcumin [33,40], we selected the curcumin concentrations of 10 μM for the following experiments.

### 2.2. Gene Expression Changes in Curcumin-Treated IPF Fibroblasts

**Figure 3:**
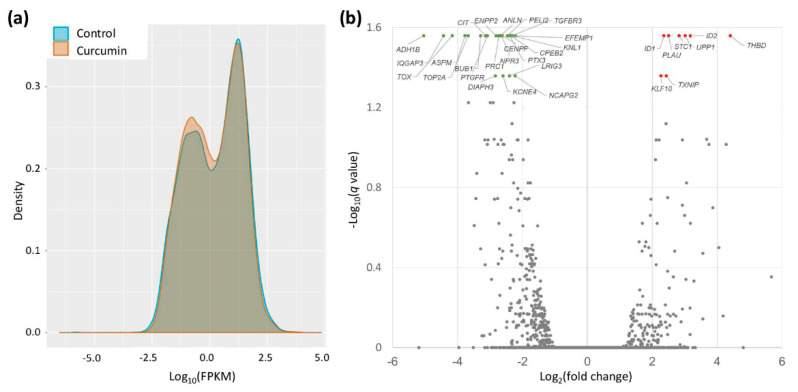
Display of gene expression data in curcumin-treated IPF fibroblasts. (**a**) The density plots illustrate smoothed frequency distribution of the fragments per kilobase of transcript per million mapped reads (FPKM) in curcumin-treated IPF fibroblasts and controls. (**b**) Volcano plot of differentially expressed genes in IPF fibroblasts treated with 10 μM curcumin versus the control. Significantly differentially expressed genes (fold change > 2 and –log_10_(*q*-value) > 1.3) are shown in green (downregulation) and red (upregulation).

The gene expression profiles in IPF fibroblasts treated with either curcumin (10 μM) or DMSO (control) were obtained by using NGS. As shown in the density plot (Figure 3a), the curcumin-treated IPF fibroblasts showed more genes with lower FPKM values compared with the controls. The volcano plot of gene expression profiles in curcumin-treated IPF fibroblasts versus the controls (Figure 3b) shows the significantly differentially expressed genes (fold change > 2 and *q*-value < 0.05). A total of 23 downregulated and 8 upregulated protein-coding genes were identified in IPF fibroblasts treated with curcumin (Table 1).

### 2.3. STRING Analysis for Protein-Protein Interaction and Biological Process of the Differentially Expressed Genes in Curcumin-Treated IPF Fibroblasts

**Figure 4:**
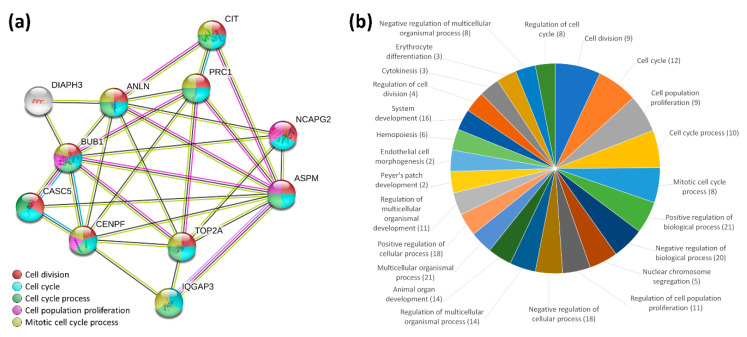
STRING analysis for protein–protein interactions and biological processes of the differentially expressed genes in curcumin-treated IPF fibroblasts. (**a**) Protein–protein interaction network analysis. The 31 differentially expressed genes (8 upregulated and 23 downregulated) were input into the Search Tool for the Retrieval of Interacting Genes (STRING) database for protein–protein interaction (PPI) network analysis. The minimum required interaction score was set to a high confidence (score = 0.700). This analysis obtained a highly interactive PPI network of 31 nodes and 35 edges, with a PPI enrichment *p*-value of 1.0 × 10^−16^. Nodes represent proteins and edges represent protein–protein associations. The disconnected nodes are not shown. Most genes in the core PPI network were related to the following biological processes: cell division (9 genes, shown in red), cell cycle (12 genes, shown in blue), cell cycle process (10 genes, shown in green), cell population proliferation (9 genes, shown in purple), and mitotic cell cycle process (8 genes, shown in yellow) (all FDR-*p* < 0.001). (**b**) Gene ontology analysis of biological processes. The biological processes with a false discovery rate-adjusted *p*-value (FDR-*p*) of <0.015 are presented. The area represents the significance of each biological process, based on −log_10_(FDR-*p*). The numbers of differentially expressed genes involved in each biological process are marked in parentheses.

The 23 significantly downregulated and 8 significantly upregulated genes in curcumin-treated IPF fibroblasts were included in the analysis for protein–protein interactions and biological processes. We uploaded these differentially expressed genes into the Search Tool for the Retrieval of Interacting Genes (STRING) database for protein–protein interaction (PPI) network analysis. We set the minimum required interaction score to the high confidence (score = 0.700). This analysis obtained a highly interactive PPI network of 31 nodes (proteins) and 35 edges (protein-protein associations), with a PPI enrichment *p*-value of <1.0 × 10^−16^ (Figure 4). Gene ontology analysis showed that most genes in the PPI network were related to the following biological processes: cell division (9 genes), cell cycle (12 genes), cell cycle process (10 genes), cell population proliferation (9 genes), and mitotic cell cycle process (8 genes) (all FDR-*p* < 0.001).

### 2.4. IPA Analysis for Cellular Functions of the Differentially Expressed Genes in Curcumin-Treated IPF Fibroblasts

**Figure 5:**
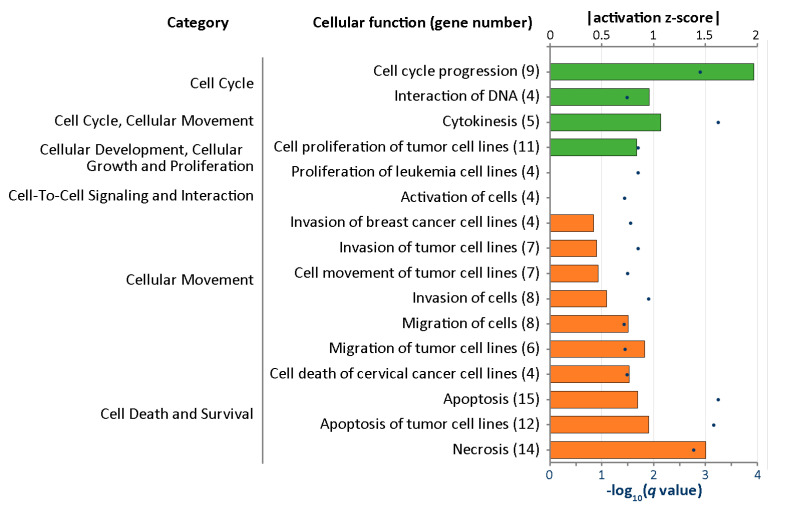
Using Ingenuity^®^ Pathway Analysis, the diseases and functions of the differentially expressed genes in curcumin-treated IPF fibroblasts were analyzed. The cellular functions with *q*-values (*p*-values adjusted by the Benjamini–Hochberg procedure) of <0.5 and available activation z-scores were selected. The numbers of differentially expressed genes involved in each cellular function are marked in parentheses. The bars represent the activation z-score (the length represents the absolute value; negative and positive scores are presented in green and orange colors, respectively) and the dark blue dots indicate −log_10_(*q*-value).

Differentially expressed genes were further analyzed with Ingenuity^®^ Pathway Analysis (IPA) to outline the most enriched functional alteration associated with curcumin treatment in IPF fibroblasts. Figure 5 shows the enriched cellular functions into which the differentially expressed genes were categorized. Downregulation of cell cycle progression was found to be the most prominent change associated with curcumin treatment in IPF fibroblast (activation z-score = −1.965, *q*-value = 0.00125); nine genes, including *ANLN*, *BUB1*, *CENPF*, *CIT*, *KLF10*, *KNL1*, *PTX3*, *TOP2A*, and *TOX*, were involved in this cellular function.

### 2.5. Differentially Expressed Genes with Potential microRNA-mRNA Interactions in Curcumin-Treated IPF Fibroblasts

Using NGS, we identified 13 significantly downregulated microRNAs and 57 significantly upregulated microRNAs associated with curcumin treatment in IPF fibroblasts (Figure 1). Using the miRmap database with a miRmap score ≥97.0 criterion, 5326 predicted targets of these differentially expressed microRNAs were identified. Combined with the lists of nine differentially expressed genes involved in cell cycle progression identified with IPA, we found a potential microRNA-mediated gene expression alteration in curcumin-treated IPF fibroblasts, namely, downregulated hsa-miR-6724-5p and upregulated *KLF10*. Using TargetScan, the prediction of *KLF10* as a target of hsa-miR-6724-5p was confirmed (site type: 8mer; context++ score: −0.51; context++ score percentile: 99).

### 2.6. Potential Effects of Curcumin on Dysregulated Genes and microRNAs in IPF Fibroblasts

**Figure 6:**
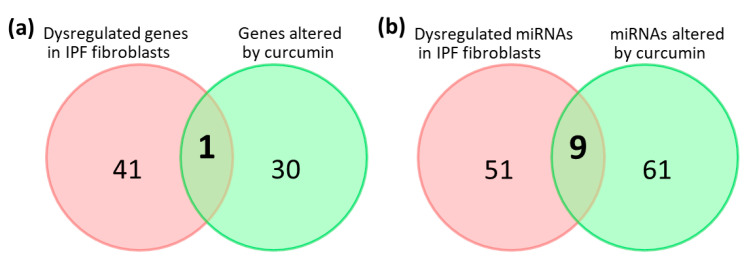
Venn diagrams showing the numbers of (**a**) altered genes and (**b**) altered microRNAs in IPF fibroblasts [6] and curcumin-treated IPF fibroblasts.

Our previous study found 42 dysregulated genes and 60 dysregulated microRNAs in IPF fibroblasts compared to normal human lung fibroblasts [6]. By combining the data, we further examined the effects of curcumin on the expressions of these genes and microRNAs. As a result, we found a common gene, *PTX3*, on both lists of dysregulated genes in IPF fibroblasts and altered genes in curcumin-treated IPF fibroblasts, which was upregulated in IPF fibroblasts compared to normal human lung fibroblasts (fold change = 2.19) and downregulated by curcumin (fold change = −5.26) (Figure 6a and Table 2). We also found nine common microRNAs on both lists of dysregulated microRNAs in IPF fibroblasts and altered microRNAs in curcumin-treated IPF fibroblasts (Figure 6b). Excluding those with changes in the same direction, we identified five microRNAs, namely, upregulated hsa-miR-3613-5p in IPF fibroblasts, which was downregulated by curcumin treatment, and downregulated hsa-miR-182-5p, hsa-miR-664a-3p, hsa-miR-4461, and hsa-miR-9-5p, which were upregulated by curcumin treatment.

## 3. Discussion

**Figure 7:**
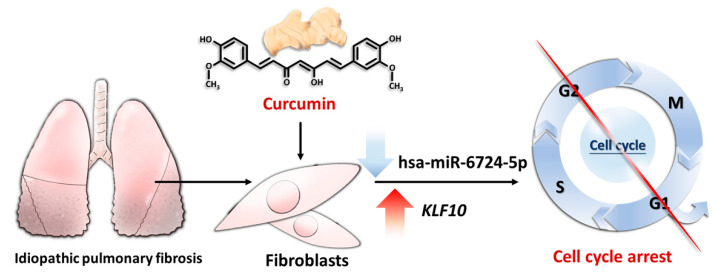
The possible effect of curcumin on IPF fibroblasts. Curcumin decreased the level of hsa-miR-6724-5p, leading to increased *KLF10* expression, resulting in cell cycle arrest.

The mechanisms underlying the pathogenesis of IPF are quite complex, possibly including oxidative stress, inflammation, enhanced proliferation, and migration of fibroblasts, and so on [1,3,5,10,21]. In the current study, we analyzed the effect of curcumin on altering the expression profiles of microRNAs and genes in IPF fibroblasts using NGS. With a bioinformatic approach, we found that curcumin treatment significantly altered the expression profiles of 31 genes, and some of them were associated with cell cycle suppression. Further analysis found that curcumin treatment decreased hsa-miR-6724-5p expression, which might contribute to increased *KLF10* expression in IPF fibroblasts (Figure 7).

Curcumin exhibits various pharmacological and biological properties, such as antioxidant, anti-inflammatory, immunomodulatory, antirheumatic, cardioprotective, hepatoprotective, renoprotective, neuro-protective, antimicrobial, antineoplastic, and hypoglycemic effects, and has been therefore extensively studied for its effects in various diseases [41,42,43]. Its potential to prevent and treat diabetes mellitus was demonstrated in some in vivo studies, showing effects in decreasing insulin resistance, improving β-cell functions, and preventing β-cell death [41]. Some studies also found potential in treating intestinal inflammatory diseases via decreasing inflammation, improving intestinal barrier functions, and altering the intestinal microbiome [44]. The potential antiaging effects of curcumin were also reported [45]. Curcumin exhibits anticancer activities via various mechanisms [46], for example, via curcumin-induced apoptosis in prostate cancer cell lines [47]. Curcumin inhibited the gene expression of Cdc25c, CDK1, and cyclin B1 and promoted the gene expression of Wee1 in colon cancer cells, causing cell cycle arrest at the G2/M phase [48]. Curcumin could induce autophagy and promote apoptosis of glioblastoma cells [49]. An in vitro study of hepatocellular carcinoma revealed that phthalate promoted metastasis and the formation of cancer stem cells, and curcumin could suppress these effects [26].

Some efforts were made to investigate the potential effects of curcumin in the treatment of pulmonary fibrosis. A previous study found that curcumin inhibited proliferation of lung fibroblasts from normal and IPF patients via cell cycle arrest at the G0/G1 phase [33]. In line with this finding, we found that curcumin might alter gene expression profiles to suppress cell cycle progression in IPF fibroblasts. In addition to proliferation inhibition, curcumin also inhibited TGF-β-dependent myofibroblast differentiation via inhibition of TGF-β-induced phosphorylation of Smad2/3 and ERK1/2, as well as inhibited collagen secretion from IPF fibroblasts [33]. Another study showed that curcumin inhibited the activation of genes associated with myofibroblast activation and proliferation, inhibited migration, induced apoptosis, and induced oxidative stress in pulmonary fibroblasts [50]. The antifibrotic effects of curcumin in pulmonary fibroblasts were also attributed to overexpression of cathepsins K and L [51], whereas another study showed that curcumin inhibited TGF-β1-dependent myofibroblast differentiation of pulmonary fibroblasts via peroxisome proliferator-activated receptor γ (PPARγ)-driven upregulation of cathepsins B and L [25]. Furthermore, other rat studies demonstrated the effect of curcumin in attenuating lung fibrosis induced by amiodarone [52], paraquat [53], and radiation [54].

Alteration of the extracellular matrix (ECM) is a major pathogenic mechanism of IPF, and fibroblasts are key effector cells of this process [55]. The current available medications for IPF, including nintedanib and pirfenidone, have heterogenous and pleotropic effects, such as anti-inflammation, inhibition of proliferation, migration, and differentiation of fibroblasts, and modulation of the secretion of ECM proteins [19,24,56,57,58,59], with the regulation of ECM being one of the most important pharmacological mechanisms. Both nintedanib and pirfenidone prevent phenotype alteration of fibroblasts induced by conditioned matrix of IPF [55]. However, in the current study, we did not find differentially expressed genes related to the regulation of ECM in curcumin-treated IPF fibroblasts.

Krüppel-like factor 10 (*KLF10*) belongs to the Krüppel-like transcription factors (KLFs) family, which is made up of 15 zinc-finger proteins able to control cell proliferation and differentiation [60]. Previously named transforming growth factor (TGF)-β-inducible early gene-1 (*TIEG* or *TIEG1*), *KLF10* is a primary response gene for TGF-β [61]. Several studies demonstrated the effect of KLF10 as a tumor suppressor via activation of p21 transcription, a well-known inhibitor of the cell cycle via CDK/cyclin inhibition and cell cycle arrest [62]. *KLF10* may mimic the antiproliferative and proapoptotic effects of TGF-β1 on various tumor cells [63,64]. Being a substrate of CDK2/cyclin E, its stability is regulated by CDK2-dependent phosphorylation; this also suggests a strong association between KLF10 and cell cycle progression [63]. *KLF10*, which is induced by a nerve growth factor, suppressed cell cycle progression of pheochromocytoma cell line PC12 [65]. *KLF10* was also found to be an effective repressor of myoblast proliferation via repression of *FGFR1* promotor activity [66]. In a study using a rat model of ischemia–reperfusion injury, upregulation of *KLF10* was associated with vascular endothelial cell arrest in the G1 phase and promotion of apoptosis [67]. *KLF10* also plays a role in the protection against oxidative stress and in myofibroblast-like conversion in human skin fibroblasts [61]. As *KLF10*-deficient mice showed increased pulmonary inflammation, KLF10 might control fibrosis by negatively regulating inflammation [68]. Although the roles of *KLF10* in regulating the cellular physiology of pulmonary fibroblasts remain largely unknown, the curcumin-induced upregulation of *KLF10* might provide potential targets to reverse pulmonary fibrosis, and therefore deserve further study.

By combining the data of the current study with our previous study, we found a common gene, *PTX3*, which was upregulated in IPF fibroblasts (vs. normal human lung fibroblasts) and downregulated by curcumin. *PTX3* encodes pentraxin 3, also known as tumor necrosis factor-inducible gene 14 (*TSG-14*) protein, which regulates multiple aspects of inflammation. The increased serum level of pentraxin 3 was associated with the disease severity of systemic sclerosis [69]. In a mice study of asthma, exogenous pentraxin 3 promoted both eosinophilic and neutrophilic airway inflammation [70]. In a clinical trial including 38 episodic migraine patients, curcumin supplementation significantly reduced the serum level of pentraxin 3, suggesting a potential inhibitory effect of curcumin on *PTX3* gene expression [71]. The role of *PTX3* in the effects of curcumin on IPF fibroblasts deserves further study.

The poor bioavailability of curcumin is always a major limitation to its wide application [29]. In a mouse study, intraperitoneal administration of curcumin reduced bleomycin-induced lung injury, whereas oral-route administration allowed a significantly lower plasma curcumin concentration to be reached and showed no significant effect in reducing bleomycin-induced lung injury [33]. In previous clinical trials of curcumin, various formulations, including liposomal encapsulation, emulsions, nanoparticles, powder, capsules, and tablets, were used [43]. We believe that the parenteral route, including inhalation and intravenous routes, might be the preferred route to deliver curcumin as a treatment of IPF, with further study still warranted. In a recent rat study, inhalation of curcumin large porous microparticles showed promising effects in attenuating bleomycin-induced lung fibrosis [29].

This study demonstrated several limitations. Firstly, the cells used in this study were the primary cells from a single IPF patient. Because fibroblasts from several IPF patients may have marked heterogeneity in the baseline gene expression profiles and the response to stimuli, our findings might not be able to be generalized to other IPF patients. However, the findings of this exploratory study might provide a scientific basis for understanding the pathogenic mechanisms and developing novel treatments in the future. Secondly, the results of the bioinformatic analyses in the present study were not validated. Further in vitro and in vivo studies are warranted to confirm our findings. Thirdly, the number of differentially expressed genes was relatively low. Although the numbers of differentially expressed genes and interesting pathways could have been increased by lowering the stringency, we selected the most significant findings in this study. Fourthly, we only assessed the transcriptomic changes in IPF fibroblasts treated with a single concentration of curcumin, which may not reflect the changes in normal human lung fibroblasts treated with curcumin. While curcumin showed similar, yet milder, inhibitory effects on cell viability/proliferation in a dose-dependent manner in normal human lung fibroblasts as those observed in IPF fibroblasts, the curcumin-related transcriptomic changes might be similar in pulmonary fibroblasts to either IPF patients or normal subjects. Finally, in addition to fibroblasts, airway epithelial cells, especially type II alveolar epithelial cells, are also key drivers in the pathogenesis of IPF [72]. Whether our findings of pulmonary fibroblasts remain stationary in the pulmonary microenvironment, containing airway epithelial cells and other cells, deserves further investigation.

## 4. Materials and Methods

### 4.1. Study Design and Analysis Strategy

Figure 1 shows a flowchart of the study design and analysis strategy. IPF fibroblasts were treated with either 10 μM curcumin or 0.1% dimethyl sulfoxide (DMSO) (control) for 48 h. Then, the harvested RNAs were sequenced for microRNA and mRNA expression profiles using NGS platforms. We focused on protein-coding genes and selected genes with significantly differential expressions for further analyses. We used bioinformatic tools, including the Search Tool for the Retrieval of Interacting Genes (STRING) database [73] and the Ingenuity^®^ Pathway Analysis (IPA) database [74], to investigate the altered functions and pathways associated with curcumin treatment. Additionally, upregulated and downregulated microRNAs were analyzed with miRmap [75] for target predictions. We identified potential microRNA–mRNA interactions associated with curcumin treatment. We used a Venn diagram to determine microRNA–mRNA interactions related to apoptosis, and validated these interactions using TargetScan [76].

### 4.2. Cultures of IPF Lung Fibroblasts and Curcumin Treatment

Primary human lung fibroblasts from an 83-year-old male Caucasian IPF patient (obtained from Lonza, Walkersville, MD, USA; Catalog No. CC-7231) and normal human lung fibroblasts (NHLF; Catalog No. CC-2512) from a 67-year-old Caucasian man were incubated at 37 °C in a Fibroblast Growth Medium-2 (FGM™-2) Bulletkit™ (Lonza; Catalog No. CC-3132) containing 10 mL fetal bovine serum, 0.5 mL human fibroblast growth factor-basic (hFGF-B), 0.5 mL insulin, and 0.5 mL GA-1000, as performed in our previous studies [6,7,8]. The medium was changed every 2–3 days and the cells were channeled after distinct cell density. Curcumin (Sigma-Aldrich, St. Louis, MO, USA; Catalog No. C7727) was dissolved in dimethyl sulfoxide (DMSO) (Sigma-Aldrich) to obtain specific concentrations. Cells (the second passage) were plated in 6 cm culture plates (1 × 10^5^ cells/well) and incubated for 24 h, and were then treated with either curcumin (10, 15, or 20 μM) or the carrier solvent (0.1% DMSO) for 48 h.

### 4.3. Cell Proliferation Assay

The cell proliferation/viability was assessed using Premixed WST-1 Cell Proliferation Assay (Takara Bio Inc., Shiga, Japan), as in our previous studies [77]. Data were expressed in means with standard deviations (means ± SD). Statistical comparisons of the results were made using analysis of variance, followed by Dunnett’s test. A two-tailed *p*-value of <0.05 was considered significant.

### 4.4. Next-Generation Sequencing (NGS)

The expression profiles of microRNAs and mRNAs were assessed using an NGS platform, as described in our previous studies [6,7,8,78,79,80,81,82]. In brief, total RNA from the IPF fibroblasts was extracted with TRIzol^®^ Reagent (Thermo Fisher Scientific, Waltham, MA, USA. Catalog No. 15596018), according to the manufacturer’s instructions. Purified RNA was quantified at OD_260nm_ by an ND-1000 spectrophotometer (Nanodrop Technology, Wilmington, DE, USA) and qualitated using the Bioanalyzer 2100 (Agilent Technologies, Santa Clara, CA, USA) with an RNA 6000 LabChip® kit (Agilent Technologies). Before library preparation, the quality of RNA was ensured based on the following criteria: amount ≥ 5 μg (for transcriptome) or ≥ 2 μg (for small RNA); concentration ≥ 80 ng/μL (for transcriptome) or ≥ 200 ng/μL (for small RNA); OD_260nm_/OD_280nm_ ratio of 1.8–2.2; OD_260nm_/OD_230nm_ ratio ≥ 1.5; RNA integrity number ≥ 7 (for transcriptome) or ≥ 8 (for small RNA). The RNA library preparation and deep sequencing were carried out in Welgene Biotechnology Company (Taipei, Taiwan), according to Illumina’s official protocol.

For transcriptome sequencing, the Agilent’s SureSelect Strand Specific RNA Library Preparation Kit was used to prepare the libraries, followed by AMPure XP Beads size selection. The sequence was directly determined using Illumina’s sequencing-by-synthesis (SBS) technology on the NovaSeq 6000 Sequencing System (Illumina, Inc., San Diego, CA, USA). Sequencing data were generated by Welgene’s pipeline based on Illumina’s basecalling program bcl2fastq v2.2.0. Reads with low quality were trimmed and removed using the Trimmomatic (version 0.36) [83]. Qualified reads were then analyzed with HISAT2 (version 2.1.0) [84], a fast and sensitive alignment program for mapping NGS reads to the genome. To normalize for sequencing depth and gene length, the expression levels of genes were expressed in fragment per kilobase of transcript per million mapped reads (FPKM), which was calculated by dividing the total reads corresponding to a single fragment by the length of the fragment (in kilobase) and by the total reads in the sample (in “per million” scale) in paired-end RNA sequencing [85,86]. Genes with low expression levels (<0.3 FPKM) in either curcumin-treated or control fibroblasts were excluded. The *p*-values for differential expression were calculated by Cuffdiff (Cufflinks version 2.2.1), with the non-grouped sample using “blind mode”, whereby all samples were treated as replicates of a single global “condition” and used to build one model for a statistical test [87]. The *q*-values were *p*-values adjusted with false discovery rate (FDR) using the method by Benjamini and Hochberg [88]. Genes with >2-fold changes (the ratio of FPKM in curcumin-treated IPF fibroblasts to the controls >2) and *q*-values of <0.05 (i.e., −log_10_ (*q*-value) > 1.3) were considered to be significantly differentially expressed genes.

For small RNA sequencing, sample libraries were prepared using the TruSeq Small RNA Library Preparation Kit (Illumina) according to the manufacturer’s protocols. The 3′- and 5′-adaptors were ligated to the microRNAs. Subsequently, universal cDNA synthesis with unique molecular identifier (UMI) assignment, cDNA cleanup, library amplification, and library cleanup were performed. Libraries were sequenced on the NextSeq500 Sequencer (Illumina, Inc., San Diego, CA, USA) (75-cycle single-end read, 75SE). Sequencing data were processed with the Illumina software BCL2FASTQ v2.20. Trimmomatics (version 0.36) [83] was used to clip the 3′ adaptor sequence, trim or remove low-quality reads, and discard trimmed reads shorter than 18 nucleotides. Qualified reads were further analyzed using miRDeep2 [89] to align reads to the reference human genome downloaded from the University of California, Santa Cruz. Because microRNAs usually map to few genomic locations, only reads that mapped perfectly to the genome ≤5 times were used for microRNA detection. MiRDeep2 estimates expression levels of known microRNAs and identifies novel microRNAs. To normalize for sequencing depth, the expression levels of microRNAs were expressed in normalized read per million [rpm], which was calculated by dividing the total reads by the total reads in the sample (in “per million” scale) [85,86]. The microRNAs with low levels (<1rpm) in either curcumin-treated or control fibroblasts were excluded. The microRNAs with >2-fold changes (the ratio of rpm in curcumin-treated IPF fibroblasts to the controls >2) were considered to be significantly dysregulated.

### 4.5. STRING Database Analysis

The functional interactions between proteins are very important and complicated in cells. The STRING database (https://string-db.org/) collects and integrates this information by consolidating known and predicted protein–protein association data for many different organisms. STRING offers the information of the associations including direct (physical) and indirect (functional) interactions, which are derived from five main sources: Conserved co-expressions, high-throughput lab experiment, automated text-mining, genomic context predictions, and previous knowledge in the database [73]. All significantly differentially expressed genes were uploaded into the STRING for analysis of altered functions and pathways associated with curcumin treatment. The minimum required interaction score was set at 0.700 to reach a high confidence level. Gene ontology analysis was also performed. The biological processes with false discovery rate-adjusted *p*-values (FDR-*p*) < 0.015 were selected.

### 4.6. The Ingenuity^®^ Pathway Analysis (IPA)

To investigate the systems biology as a whole integrated system, network-based analysis is important. The IPA software (Qiagen, Redwood City, CA, USA) contains a large database with detailed and structured findings reviewed by experts. This IPA database is derived from thousands of biological, chemical, and medical researches, and provides researchers with quick searching. The IPA can also survey the analysis, integration, and recognition of genes, single nucleotide polymorphism (SNP) arrays, RNA and small RNA sequencing, proteomics, and many other biological experiments. Furthermore, IPA provides identification of associated signaling pathways, molecular interactions, upstream regulators, disease processes, and candidate biomarkers [74]. All significantly differentially expressed genes were uploaded to IPA for analysis of altered functions associated with curcumin treatment. The cellular functions with *q*-values (*p*-values adjusted by the Benjamini–Hochberg procedure) of <0.5 and available activation z-scores (representing the magnitude of significant activation) were selected.

### 4.7. miRmap Database Analysis

microRNAs can repress the expression of protein-coding genes. The miRmap (http://mirmap.ezlab.org/) is an open-source software library which provides predictions of comprehensive microRNA targets [75]. By calculating the complementary ability of microRNA–mRNA interactions, we can identify several putative target genes. The miRmap uses high-throughput experimental data from immunopurification, transcriptomics, proteomics, and polysome fractionation experiments to examine feature correlations and compare the predictive power. The prediction results provide a list of putative target genes with the miRmap score, a predictive reference value. In this study, we used an miRmap score of ≥97.0 as the criterion for selection of putative microRNA targets.

### 4.8. TargetScan Database Analysis

The TargetScan is an online database (http://www.targetscan.org) that predicts the targets of microRNAs by searching for the presence of conserved 8mer-, 7mer-, and 6mer- sites matching the seed region of each microRNA. The prediction results are ranked by the probability of conserved targeting or by the predicted efficacy of targeting [76]. The TargetScan is therefore a quite powerful resource for investigating the role of microRNAs in gene-regulatory networks.

## 5. Conclusions

In this study using an NGS and bioinformatics approach, we found that curcumin might decrease the level of hsa-miR-6724-5p, leading to increased *KLF10* expression, resulting in cell cycle arrest in curcumin-treated IPF fibroblasts. The current findings might support the potential role of curcumin in the treatment of IPF, but further in-depth study is warranted to confirm our findings.

## Figures and Tables

**Table 1:** Differentially expressed protein-coding genes in curcumin-treated IPF fibroblasts.

Gene Symbol	Curcumin 10 μM (FPKM)	Control (FPKM)	Ratio (Curcumin/Control)	*p*-Value	*q*-Value *
*ADH1B*	2.83	93.89	0.03	0.00005	0.0276
*IQGAP3*	0.45	9.71	0.05	0.00005	0.0276
*TOX*	0.72	13.08	0.06	0.00005	0.0276
*ASPM*	1.70	23.23	0.07	0.00005	0.0276
*TOP2A*	5.57	71.17	0.08	0.00005	0.0276
*BUB1*	2.59	25.37	0.10	0.00005	0.0276
*CIT*	2.23	19.76	0.11	0.00005	0.0276
*PTGFR*	7.94	67.35	0.12	0.00005	0.0276
*ENPP2*	28.62	201.09	0.14	0.00005	0.0276
*DIAPH3*	2.88	20.55	0.14	0.00010	0.0439
*PRC1*	6.26	41.46	0.15	0.00005	0.0276
*CENPF*	3.41	20.98	0.16	0.00005	0.0276
*NPR3*	8.38	52.27	0.16	0.00005	0.0276
*ANLN*	8.44	54.04	0.16	0.00005	0.0276
*KCNE4*	3.31	19.98	0.17	0.00010	0.0439
*PELI2*	4.86	26.82	0.18	0.00005	0.0276
*PTX3*	78.97	415.71	0.19	0.00005	0.0276
*LRIG3*	4.42	23.31	0.19	0.00010	0.0439
*KNL1*	2.13	10.42	0.20	0.00005	0.0276
*TGFBR3*	8.78	44.60	0.20	0.00005	0.0276
*CPEB2*	29.38	149.72	0.20	0.00005	0.0276
*NCAPG2*	5.19	24.29	0.21	0.00010	0.0439
*EFEMP1*	42.08	195.48	0.22	0.00005	0.0276
*KLF10*	77.02	16.02	4.81	0.00010	0.0439
*ID1*	154.13	30.14	5.11	0.00005	0.0276
*TXNIP*	41.05	7.59	5.41	0.00010	0.0439
*PLAU*	84.67	14.96	5.66	0.00005	0.0276
*STC1*	79.28	11.17	7.10	0.00005	0.0276
*UPP1*	80.27	9.93	8.08	0.00005	0.0276
*ID2*	68.30	7.57	9.02	0.00005	0.0276
*THBD*	25.91	1.22	21.30	0.00005	0.0276

* The *q*-values are *p*-values adjusted with false discovery rate using the method by Benjamini and Hochberg. Abbreviation: FPKM, fragments per kilobase of transcript per million mapped reads.

**Table 2:** Dysregulated microRNAs in IPF fibroblasts [6] altered by curcumin.

microRNA	IPF Fibroblasts vs. NHLF		Curcumin-Treated IPF Fibroblasts vs. Control IPF Fibroblasts	
	Fold Change	Up/Down	Fold Change	Up/Down
hsa-miR-3613-5p	2.76	up	−5.15	down
hsa-miR-182-5p	−2.01	down	3.24	up
hsa-miR-664a-3p	−2.46	down	2.14	up
hsa-miR-4461	−3.05	down	2.13	up
hsa-miR-9-5p	−3.05	down	5.23	up
hsa-miR-204-5p	−3.77	down	−2.96	down
hsa-miR-4521	3.67	up	4.08	up
hsa-miR-619-5p	2.94	up	4.27	up
hsa-miR-668-3p	2.06	up	3.79	up

Abbreviation: NHLF, normal human lung fibroblasts.
